# Cancer risks among long-standing spouses

**DOI:** 10.1038/sj.bjc.6600302

**Published:** 2002-06-05

**Authors:** K Hemminki, Y Jiang

**Affiliations:** Department of Biosciences at Novum, Karolinska Institute, 141 57 Huddinge, Sweden

**Keywords:** cancer in spouses, familial cancer, heredity, genetic epidemiology

## Abstract

We estimated risks for concordant and discordant cancers in spouses in order to quantify cancer risks from the shared environment. The study was restricted to spouses who had one or more children in common and who lived together for at least 15 years after the first child's birth. The nation-wide Family-Cancer Database was used as the source of family and cancer data. Standardised incidence ratios were calculated for concordant and discordant cancers in spouses after 50 years of age. Among the 18 cancer sites considered, only three sites, stomach, lung and bladder, showed concordant increases of cancer among spouses, standardised incidence ratios ranging only from 1.19 to 1.38. Additionally, gastric and pancreatic cancer were associated among spouses, as did many cancers which were related to tobacco smoking or human papilloma virus infection. By contrast, standardised incidence ratios of colon, rectal, renal and skin cancers showed no increases among spouses. Shared lifestyle among family members seems to explain only a small proportion of familial cancer susceptibility. Because lifestyles are likely to differ more between parents and offspring than between spouses, familial cancer risks between parents and offspring are even more likely to be due to heritable than environmental effects.

*British Journal of Cancer* (2002) **86**, 1737–1740. doi:10.1038/sj.bjc.6600302 www.bjcancer.com

© 2002 Cancer Research UK

## 

Many lines of epidemiological evidence indicate that cancer is mainly an environmental disease (Doll and Peto, 1981; IARC, 1990; Lichtenstein *et al*, 2000; Peto, 2001). During the past decade it has become increasingly clear that overweight and lack of physical activity convey a risk of cancer, which may account for 5% of all cancers in Europe (Bergstrom *et al*, 2001; IARC, 2002). Moreover, the risks at the population level caused by various infections have become better understood, and the known infections have been estimated to account for 15% of cancer world-wide, though less in Europe (Pisani *et al*, 1997; Zur Hausen, 1999). In spite of the enormous research effort on diet and cancer, the proportion of cancer attributable to diet or to any specific dietary component remains speculative. It has been estimated that at least 50%, and probably as much as 70% of cancer deaths are unavoidable among non-smokers mainly because their aetiology remains unknown (Peto, 2001).

Decades long cohabitation by spouses should tend to result in many habits and carcinogenic exposures being similar. Interest in disease among spouses earlier focused on sexually transmitted diseases and the effects of passive smoking (IARC, 1995; Hackshaw, 1998; Hemminki *et al*, 2000a; Hemminki and Dong, 2000a). Besides assessing life-style factors and cancer risks, they can point to the environmental contribution to the familial aggregation of cancer, and they thus help to apportion heritable effects (Hemminki *et al*, 2001a,d). The studies from the Swedish Family-Cancer Database have shown limited spouse concordance, affecting mainly the sites of known environmental carcinogens (Hemminki and Dong, 2000b; Hemminki *et al*, 2001a). However, in the previous studies the length of cohabitation between the spouses was not considered, nor were any adjustments for socio-economic status carried out. We address these shortcomings here in a study of the 2001 update of the Family-Cancer Database, covering 10.2 million individuals and over one million tumours (Hemminki *et al*, 2001c). In addition to concordant cancers in spouses, a systematic analysis of discordant cancers was also carried out.

## METHODS

The Swedish Family-Cancer Database includes persons born after 1932 with their biological parents (Hemminki *et al*, 2001c) together with cancers retrieved from the nationwide Swedish Cancer Registry for the years 1958 to 1998. Additionally, residential and socio-economic data were included from national censuses, carried out in 1960, 1970, 1980 and 1990. A four-digit diagnostic code according to a modified version of the seventh revision of the International Classification of Diseases (ICD-7) was used. The following sites were examined collectively: ‘upper aerodigestive tract’, lip, mouth and pharynx (codes 140, 141, 143–148) and leukemia (204–207), polycythemia vera (208) and myelofibrosis (209). Skin cancer only included squamous cell carcinoma; basal cell carcinoma is not registered in the Cancer Registry.

Spouses were defined as the parents of the woman's first child, and they had to live in a shared address in at least two subsequent decennial censuses; thus the minimal cohabitation was 15 years by average. Even though data were available on the marital status, the above definition was preferable because many couples live together without being married. Follow-up was started at the age of 50 years, to allow latency time from the start of cohabitation. Standardized incidence ratios (SIRs) were calculated as the ratio of observed (O) to expected (E) number of cases. The expected numbers were calculated from site-, age-, period (10-year bands), area (three areas, three large cities, south Sweden and the rest), socio-economic status (manual workers, ‘intermediate’ workers, professionals and others) – and sex-standardized rates (Esteve *et al*, 1994). SIRs for women were additionally adjusted for parity (one or more; all women were parous) and age at first birth (<20, 20–29 or more years). SIRs were calculated for a spouse when the partner, proband, presented with the same, concordant, or another, discordant cancer. The reference group was spouses without cancer. Confidence intervals (95% CI) were calculated assuming a Poisson distribution (Esteve *et al*, 1994).

In analysis of discordant sites for cancers occurring in both genders, four comparisons were possible, i.e., (1) gastric cancer in husband by pancreatic cancer in wife; (2) pancreatic cancer in husband by gastric cancer in wife; (3) gastric cancer in wife by pancreatic cancer in husband; (4) pancreatic cancer in wife by gastric cancer in husband. The number of affected pairs is identical in (1) and (3), and also in (2) and (4); the calculated SIRs were often quite similar. However, (1) and (2), and also (3) and (4) were entirely independent analyses, and the results in this study have only been presented if some consistency was found in more than one type of analysis. This was a useful safeguard against false positive findings (Dong and Hemminki, 2001).

## RESULTS

A total of 71 020 couples presented with a concordant or discordant cancer after age 50 years, who fulfilled the entrance criteria for the study of being parents to the first child of the women and residing in a shared address at least through two consecutive censuses after the first childbirth. In Table 1

**Table 1:**
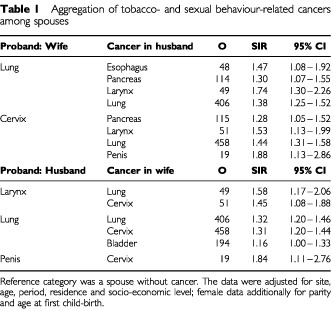
Aggregation of tobacco- and sexual behaviour-related cancers among spouses

, we show usefulness of the approach, by comparing cancer sites among spouses where increased risks should be expected due to shared smoking and sexual habits. When wives were probands and presented with lung cancer, husbands had increased risks of oesophageal (SIR 1.47), pancreatic (1.30), laryngeal (1.74) and lung (1.38) cancers. Cervical cancer was associated with pancreatic (1.28), laryngeal (1.53), lung (1.44) and penile (1.88) cancers in the husband. When husbands presented with a larynx cancer, their wives had an excess of lung (1.58) and cervical cancer (1.45). Lung cancer in husbands was associated with lung (1.32), cervical (1.31) and bladder (1.16) cancer in wives. Penile cancer was associated with cervical cancer in the wife (1.84).

Risks for spouses for concordant cancers are shown in Table 2

**Table 2:**
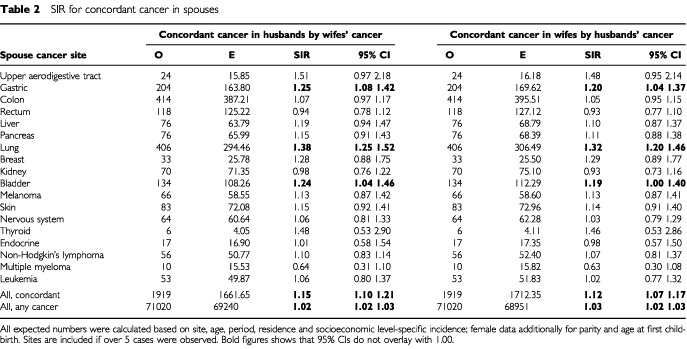
SIR for concordant cancer in spouses

for sites where more than five pairs were recorded. Cancer at three sites was increased for husbands by cancer in wives: gastric (SIR 1.25), lung (1.38) and bladder (1.24) cancers. Because of the large number of cases at these sites, all concordant cancers were increased to 1.15, but all cancers, including discordant ones, were increased only to 1.03. Results for wives were similar, although the SIRs were somewhat lower: 1.20 for gastric, 1.32 for lung and 1.19 for bladder cancers.

We analysed systematically associations between all discordant sites. In addition to the smoking and sexual behaviour related increases, such as those shown in Table 1, the only other consistent ones, exceeding SIR 1.10 and showing statistical significance, linked stomach and pancreatic cancers, as shown in Table 3

**Table 3:**
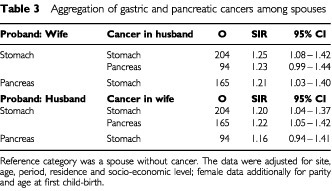
Aggregation of gastric and pancreatic cancers among spouses

. Significant increases were for husbands' gastric cancer by wives' pancreatic cancer (1.21) and for wives' pancreatic cancer by husbands' stomach cancer (1.22). A few other significant associations between the spouses' discordant cancer sites were observed but because they were not confirmed in analysis when the sites were reversed (see last paragraph of Subjects and Methods), the data are not shown.

## DISCUSSION

The present analysis focused on the possible cancer risk factors that could be observed from long-standing spouses with at least 15 years of cohabitation, by definition. The main finding was that spouses did not share cancer risks, with the exception of a few associations that are discussed below. Assuming that the couples share at least some dietary habits and features of life-style, these do not appear to influence cancer risks. This finding was perhaps most remarkable for colorectal cancer for which diet is considered an important factor (Fund, 1997). A weakness of the present study is that we have no dietary data and the study is ecological in this regard. Our data cannot take into account the differences in dietary habits of spouses that persisted through long periods of cohabitation. On the other hand, these data are consistent with the thrust of the migrant studies that suggest that the main shift of the cancer pattern in the new host country takes place between generations one and two (McCredie, 1998; Parkin and Iscovich, 1997). The data on immigrants to Sweden are quite clear-cut on this point. The first generation immigrants who have entered the country in adult age follow a cancer pattern resembling the country of origin, while their Swedish-born children have perfectly adjusted to the Swedish cancer experience (Hemminki and Li, 2002a,b; Hemminki *et al*, 2002b).

Multiple comparisons are a problem in this kind of study but we could address this by comparing two sites in two ways, as explained at the end of Methods. Among discordant sites that showed increased risks among spouses, sexual and smoking habits were the likely reason for the associations found in Table 1. Human papilloma virus (HPV) infection is the main etiological factor for cervical cancer and it is also strongly indicated in penile cancer (Dillner *et al*, 2000; Zur Hausen, 2000). The SIRs between these two cancers were the highest noted for the whole study, over 1.8. The strong association of tobacco- and HPV-related cancers has been a common finding in the Family-Cancer Database, even across two generations (Hemminki *et al*, 1999, 2001b). Such associations are likely to be an indication of a life-style, for which tobacco smoking, alcohol consumption, sexual promiscuity and divorce are some common denominators (Hemminki and Jiang, 2002b; Kvikstad *et al*, 1994).

The concordant sites for which the spouses shared risk were stomach, lung and bladder, all with modest SIRs ranging from 1.19 to 1.38. Tobacco is surely responsible for the concordance of lung and bladder cancer. For stomach cancer, up to 60% of the cases are attributed to *Helicobacter pylori* in developed countries and this infection tends to run in families (Bevan and Houlston, 1999; Hamilton and Aaltonen, 2000; Hemminki and Jiang, 2002a; Pisani *et al*, 1997). Even though the infectivity of *Helicobacter* may not be high among adults, it is still the main aetiological candidate (Goodman and Correa, 2000); however dietary factors, such as vitamin deficiencies and salty food items, may also contribute (Ekstrom *et al*, 2000). There was an association between gastric and pancreatic cancers in spouses. Among the known or suggested environmental causes of pancreatic cancer, tobacco smoking, obesity and the resulting diabetes, high caloric intake and alcohol consumption are likely to be shared to some degree by spouses but none of them are important in stomach cancer (Weiderpass *et al*, 1998).

The present results on spouses have implications for the interpretation of familial risks of cancer, which are usually much higher than those found in the present study (Dong and Hemminki, 2001; Goldgar *et al*, 1994; Risch, 2001). Because lifestyles are likely to differ more between parents and offspring than between spouses, familial cancer risks between parents and offspring are more likely to be due to heritable rather than environmental effects. As a reservation, it needs to be considered that childhood and youth may be the most vulnerable period for carcinogenesis. Yet the present results suggest that, with the possible exception of lung cancer, the reported familial risks in cancer that occur in both genders are mainly due to heritable factors, many of which are yet unknown (Hemminki *et al*, 2001a). In summary, the present analysis on cancer risks among spouses showed no associations, which could not be explained by known risk factors, with the exception of the association between gastric and pancreatic cancers.
